# Ultralight boron nitride aerogels via template-assisted chemical vapor deposition

**DOI:** 10.1038/srep10337

**Published:** 2015-05-15

**Authors:** Yangxi Song, Bin Li, Siwei Yang, Guqiao Ding, Changrui Zhang, Xiaoming Xie

**Affiliations:** 1 Science and Technology on Advanced Ceramic Fibers and Composites Laboratory, College of Aerospace Science and Engineering, National University of Defense Technology, 109 Deya Road, Changsha 410073, PR China; 2 State Key Laboratory of Functional Materials for Informatics, Shanghai Institute of Microsystem and Information Technology, Chinese Academy of Sciences, 865 Changning Road, Shanghai 200050, PR China

## Abstract

Boron nitride (BN) aerogels are porous materials with a continuous three-dimensional network structure. They are attracting increasing attention for a wide range of applications. Here, we report the template-assisted synthesis of BN aerogels by catalyst-free, low-pressure chemical vapor deposition on graphene-carbon nanotube composite aerogels using borazine as the B and N sources with a relatively low temperature of 900 ^°^C. The three-dimensional structure of the BN aerogels was achieved through the structural design of carbon aerogel templates. The BN aerogels have an ultrahigh specific surface area, ultralow density, excellent oil absorbing ability, and high temperature oxidation resistance. The specific surface area of BN aerogels can reach up to 1051 m^2^ g^−1^, 2-3 times larger than the reported BN aerogels. The mass density can be as low as 0.6 mg cm^−3^, much lower than that of air. The BN aerogels exhibit high hydrophobic properties and can absorb up to 160 times their weight in oil. This is much higher than porous BN nanosheets reported previously. The BN aerogels can be restored for reuse after oil absorption simply by burning them in air. This is because of their high temperature oxidation resistance and suggests broad utility as water treatment tools.

Boron nitride (BN), an analogue of carbon, with high temperature oxidation resistance^1^,^2^, low dielectric constant 3,4,5, wide bandgap^6^ and optical absorption and photoluminescence at deep ultraviolet (DUV) area^7^,^8^, is leading to a wide variety of applications as structural, thermal, electronic and optical materials. BN aerogels^9^,^10^ are special BN-based porous materials with continuous three-dimensional (3D) network structure, ultralow density and high specific surface area. They are attracting great attention for their potential applications as catalyst supports^11^,^12^, gas storage tools^13^,^14^,^15^,^16^,^17^,^18^ and water treatment tools^19^,^20^, etc. They are particularly fascinating because ultralight carbon aerogels with mass density lower than that of air (1.29 mg cm^−3^) have been reported^21^,^22^. Considering that BN has a similar lattice structure with carbon, ultralight BN aerogels with mass density less than air are also possible. However, proper synthesis routes and advanced structural design are still among the great challenges for ultralight BN aerogels.

Various routes have been used for the synthesis of BN-based porous materials. Typical routes include the direct chemical reaction method^12^,^19^,^23^, elemental substitution method^10^,^24^,^25^ and template-assisted method^20^,^26^,^27^,^28^,^29^,^30^,^31^. Of these, the direct chemical reaction method usually produces BN-based porous materials without a continuous 3D network structure, and thus it is not suitable for the synthesis of BN aerogels. The elemental substitution method offers an in situ route for BN-based materials; however, the synthesis temperature is usually very high (over 1600 °C). Template-assisted methods allow the BN materials to copy the structure of the templates with subsequent removal of the template. Synthesis temperatures for this method are usually much lower than that of elemental substitution because the templates do not take part in the reaction and are easy to remove after the growth of the BN. Moreover, due to their special preparation procedure, the size, morphology, and properties of the templates usually have marked effects on the BN products. For example, BN foam with an ultralow mass density (1.6 mg cm^−3^) has been reported[31] with Ni foam employed as a catalyst and template. However, this route cannot be applied for the synthesis of BN-based porous materials without a catalyst—the commercial Ni template restricts the size and morphology of the BN foam.

We use carbon aerogel templates to create BN aerogels via a template-assisted and catalyst-free route. Carbon aerogels (graphene-carbon nanotubes composite aerogels) were prepared in house and selected as templates for the BN aerogels. The size and morphology of carbon templates can be easily controlled using a literature method^21^. The carbon templates in our work have a continuous 3D stacking network structure of carbon nanotubes (CNTs) that are coated with graphene. They are super-elastic and resistant to fatigue^32^. Template-assisted methods allow the BN aerogels to copy the structure of the carbon aerogel templates, and thus it is important to find an effective process for the BN to grow on carbon substrates. Chemical vapor deposition (CVD) is a promising and controllable process for the growth of thin films^33^,^34^,^35^. Furthermore, as our BN aerogels follow the structure of the starting Graphene/CNT templates via the template-assisted CVD, the CNTs in starting Graphene/CNT templates would affect the pore size distribution of the resulting BN aerogels, and affect their oil absorption properties as a result. Compounds with a 1:1 B/N stoichiometry are often selected as h-BN precursors for CVD, and borazine (B_3_N_3_H_6_) could be a promising choice because it might produce BN and hydrogen both of which are environmentally friendly.

Herein, we propose a template-assisted and catalyst-free CVD route for BN aerogels using borazine as the BN precursor. Borazine was synthesized and purified according to our previous reports^36^,^37^. The BN coatings formed on the internal surface of the carbon aerogel templates via low pressure CVD at the relatively low temperature of 900 °C. Then, the BN aerogels were achieved with a removal of carbon aerogel templates in flowing oxygen at 600 °C. The resulting BN aerogels have a continuous 3D network structure, which is totally different from the structure of traditional BN-based porous materials. Furthermore, the BN aerogels are of high specific surface area and low density both of which are controllable across a large regime. The specific surface area of BN aerogels can reach up to 1051 m^2^/g, which is 2-3 times larger than that of BN aerogels reported previously (350 m^2^ g^−1^[9] and 431 m^2^ g^−1^[10]). The mass density can be as low as 0.6 mg cm^−3^—a value much lower than that of air (1.29 mg cm^−3^) and ultralight BN foam (1.6 mg cm^−3^[31]). The BN aerogels are highly hydrophobic and can absorb oil up to 160 times their own weight. This is much larger than that of porous BN nanosheets in previous reports (33 times [20]).

## Results

### Photograph, structure, and morphology of aerogels

**Figure 1:**
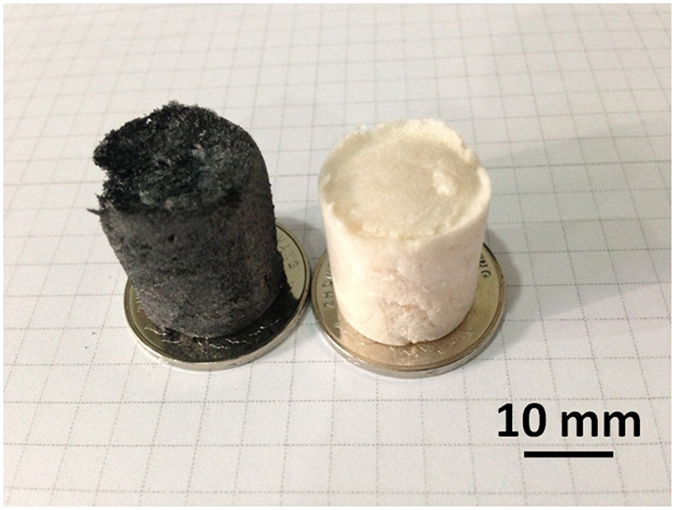
Photograph. Carbon aerogel template (left) and BN aerogel (right). The aerogels show significant color change from black to white, which indicates an obvious change in their chemical composition. The BN aerogel maintains the cylindrical shape of the carbon aerogel template.

Photographs are simple, intuitive, and offer detailed information about a sample. Figure 1 shows the photograph of a carbon aerogel template and an as-synthesized BN aerogel. Though the BN aerogel maintains the structure of the carbon aerogel (see XRD spectra of Figure S1 in the Supplementary Information), the aerogels change from black to white indicating a total conversion in the chemical composition. The BN aerogel maintains the original size and cylindrical shape of carbon aerogel templates.

**Figure 2:**
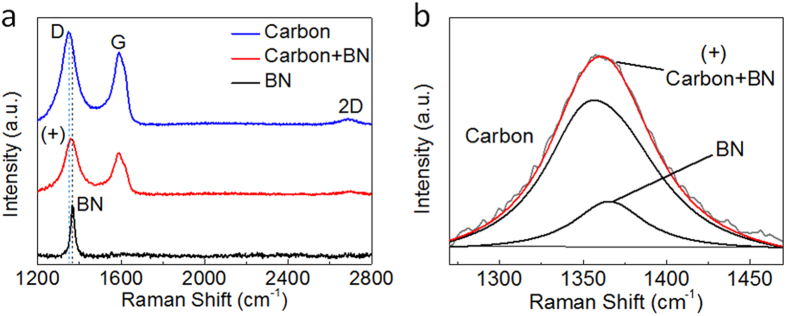
Raman spectra. (**a**) Raman spectra of a carbon areogel template, a BN/carbon aerogel and a BN aerogel. (**b**) The (+) peak of the BN/carbon aerogel in panel **a** is attributed to two parts—the D peak of carbon and the E_2g_ vibration peak of BN as fitted by Gaussian curves.

Raman spectra provide useful information about the lattice vibration modes of the aerogels. Figure 2 shows the Raman spectra of a carbon aerogel template, a BN/carbon aerogel, and a BN aerogel. The Raman peak centered at 1366 cm^−1^ was observed in the BN aerogel (Fig. 2(a)) and indicates the existence of BN. This is also confirmed by the FT-IR result in Figure S2 of the Supplementary Information. The carbon-based materials show strong D and G peaks in Fig. 2(a) as previously reported for carbon aerogels^10^. The (+) peak of the BN/carbon aerogel in Fig. 2(a) were fitted with Gaussian curves as shown in Fig. 2(b). This indicates the combination of BN and carbon in the intermediate product BN/carbon aerogel.

**Figure 3:**
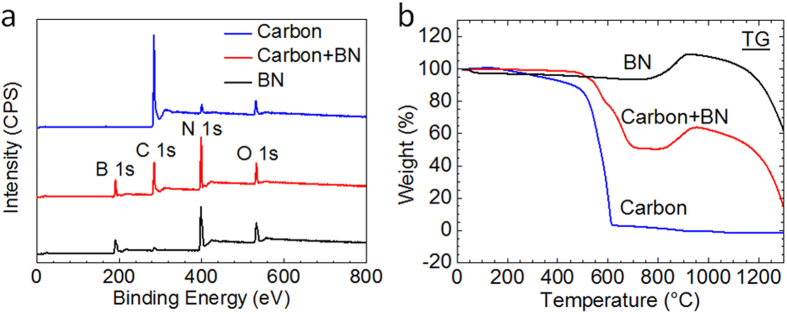
XPS and TG measurements of aerogels. (**a**) XPS spectra of a carbon aerogel template, a BN/carbon aerogel and a BN aerogel. (**b**) The TG analysis for aerogels in O_2_/N_2_ (1:4) from 0 to 1300 °C.

We used X-ray photoelectron spectra (XPS) to characterize the elemental composition and chemical state of the aerogels (Fig. 3(a)). Figure 3(a) shows that the starting graphene/CNT aerogels are composed of carbon (C), oxygen (O) and nitrogen (N). The test report along with the XPS measurements also offered the elemental ratios. According to the test report, the C, O, and N of the starting graphene aerogels were 88.21%, 5.85%, and 5.94%, respectively. As a result, the C/O ratios of the starting graphene aerogels are 88.21/5.85. The weak N 1 s peak of the as-received carbon aerogel is presumed to originate from its preparation procedure and the reduction of graphene oxide with hydrazine vapor. The O 1 s peak for all the three aerogels may originate from moisture absorption or mild oxidation, which are inevitable during storage and transfer. The binding energies of B 1 s and N 1 s for the BN aerogel are 190.9 and 398.1 eV, respectively. The B/N atomic ratio is 1.02 and indicates the nearly stoichiometric composition of the synthesized BN aerogels.

We next used TG analysis for aerogels in O_2_/N_2_ (1:4) from 0 to 1300 °C to compare the oxidation resistance of the aerogels (Fig. 3(b)). The TG results show that the BN aerogel have thermal stability over 800 °C under oxidizing conditions. The carbon aerogel shows weight loss as a result of oxidation, with nearly 0% weight residue over 600 °C. The BN aerogel exhibits slight weight increases for slight oxidation of BN over 800 °C. There was gradual weight loss for the sublimation of B_2_O_3_ over 900 °C, but over 60% weight residue at 1300 °C. These results indicate that the BN aerogels have much stronger oxidation resistance at high temperature than carbon aerogels. For example, both carbon aerogels and BN aerogels can be used as catalyst carriers at room temperature but over 600 °C the carbon aerogels fail while BN aerogels do not.

**Figure 4:**
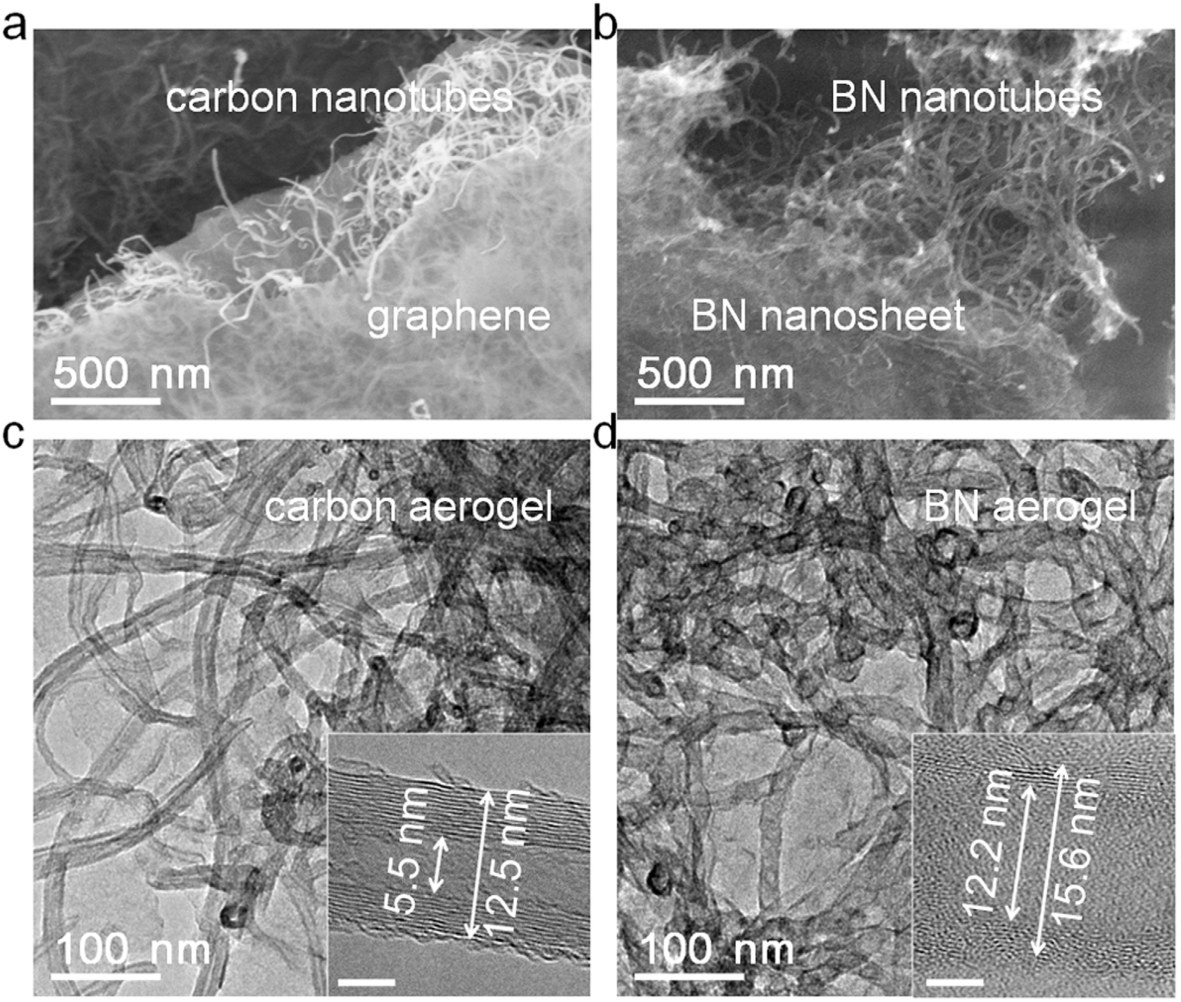
SEM and TEM images of carbon areogel template (a,c) and BN aerogel (b,d). SEM images in **a**,**b** show that the aerogels have nanotube structures coated by the nanosheets. TEM images in **c**,**d** exhibit the tubular morphologies of the aerogels at a higher magnification. The insets of **c**,**d** show representative high definition images of a carbon nanotube and a BN nanotube with arrows indicating their inside and outside diameters, respectively. Scale bars are 5 nm for insets in **c**,**d**.

SEM and TEM were used to observe the morphologies of the aerogels. The SEM images show that both the carbon aerogel and the BN aerogel have 3D network structures of nanotubes coated with nanosheets (Fig. 4(a,b)). The atomically thin graphene and BN nanosheets can barely be recognized in the TEM images in Fig. 4(c,d), but the nanotubes are obvious. The insets of Fig. 4(c,d) show a typical carbon nanotube with an outside diameter of 12.5 nm as well as a typical BN nanotube with an inside diameter of 12.2 nm, respectively. The values are quite similar because of the templated-assisted growth of BN on carbon aerogels—the carbon templates are removed in oxygen. In addition, the BN nanotube tends to follow the layered structure of the carbon nanotube substrate. The similar structure between the BN and the carbon promotes the nucleation of BN on carbon, which has been reported previously^38^,^39^. However, the BN nanotube shows a discontinuous and imperfect layered structure indicating that it has relatively lower crystallinity than the carbon nanotube. This is because we used a catalyst-free and low temperature CVD environment in our work.

**Figure 5:**
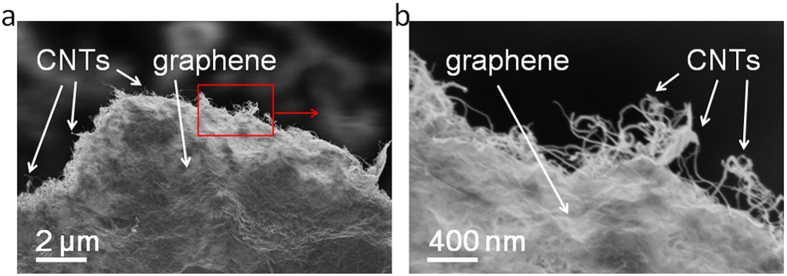
SEM images of a graphene/CNT aerogel. (**a**) Lower magnification. (**b**) Higher magnification.

**Figure 6:**
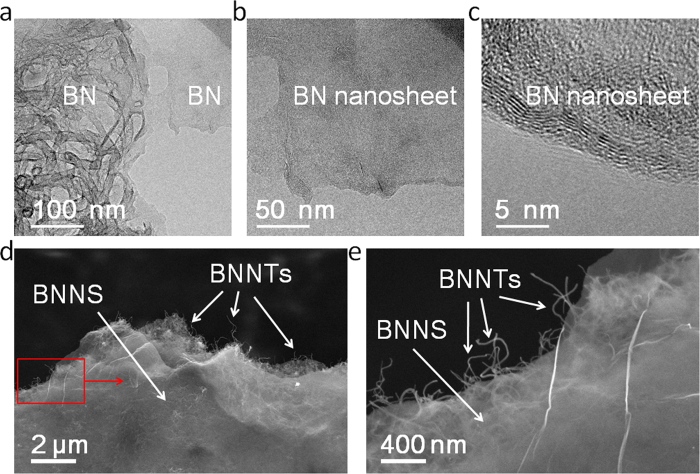
TEM and SEM images of BN aerogels. (**a**) TEM image of a BN aerogel with nanotubes and nanosheets. (**b**) TEM image of the BN nanosheet. (**c**) HRTEM of the edge of the BN nanosheet. (**d**) SEM image of a BN aerogel at lower magnification. (**e**) SEM image of the BN aerogel at Higher magnification.

The SEM data show the graphene sheet size. Figure 5 illustrates the SEM morphologies of a graphene/CNT aerogel. The aerogel has a 3D network of carbon nanotubes (CNTs) coated with graphene, and the graphene sheet size of the starting graphene/CNT aerogels was about 5–10 μm (Fig. 5(a)). Moreover, to understand the average number of layers in the BN aerogels, we used TEM to see the edge of the BN nanosheets in the BN aerogels, as shown in Fig. 6(a,b,c). The HRTEM results show that the average number of layers in the BN aerogels is about five to six, as indicated by the parallel lines near the edge of the BN nansheet in Fig. 6(C). The result is quite consistent with the HRTEM result of the BN nanotubes in Fig. 4(d). The SEM data illustrate the sheet size of a single BN sheet. Figure 6(d,e) shows the SEM morphologies of a BN aerogel—the aerogel had a 3D network structure of BN nanotubes (BNNTs) coating the BN nanosheets (BNNS). The sheet size of single BN sheet was about 5–10 μm (Fig. 6(d)).

### Porosity of aerogels

**Figure 7:**
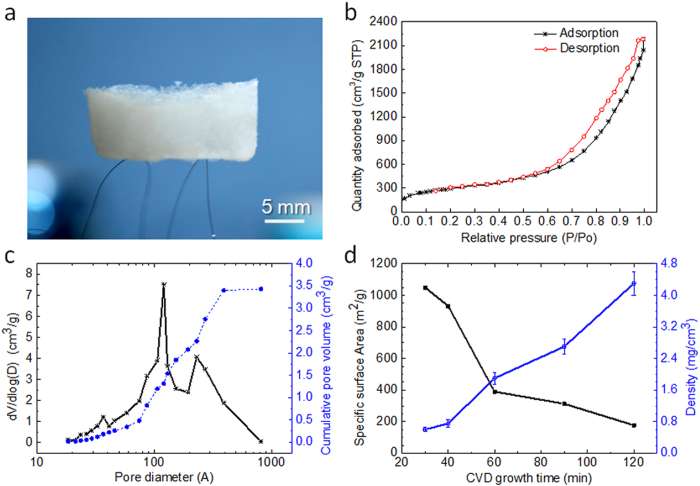
Ultralight BN aerogels with a high specific surface area. (**a**) Photograph for a BN aerogel of 1.8 cm^3^ on human hair with a mass density of 0.6 mg cm^−3^ and a high BET specific surface area of 1051 m^2^ g^−1^. (**b**) The N_2_ adsorption/desorption isotherms of the BN aerogel at 77 K. (**c**) Pore size distribution of the BN aerogel given by the BJH method including peaks at 12.1 nm and 23.1 nm. (**d**) BET surface area and density of BN aerogels as functions of the CVD growth time, also illustrating that the BN aerogel in **a** was synthesized with a CVD growth of 30 min.

One of our long-term research goals has been to develop ultra-lightweight BN materials with a mass density lower than air (1.29 mg cm^−3^). Figure 7 shows the ultra-lightweight BN aerogels with high specific surface area. As shown in Fig. 7(a), a BN aerogel with a volume of 1.8 cm^3^ can easily be supported by human hair. The aerogel has an ultralow mass density of 0.6 mg cm^−3^, which is much lower than that of air (1.29 mg cm^−3^) and ultra-light BN foam (1.6 mg cm^−3^)[31]. The density was calculated by the mass content except (minus entrapped air) divided by the volume of the aerogel. Interestingly, as a result of its relatively lower mass density, this aerogel is more transparent than the BN aerogel shown in Fig. 1 (4.0 mg cm^−3^).

Nitrogen adsorption isotherms and pore size distributions of the aerogel were measured at 77 K (Fig. 7(b)). The Brunauer-Emmett-Teller (BET) specific surface area was calculated from the nitrogen adsorption data at pressures ranging from 0.05 to 0.30. The BN aerogel exhibits a high BET specific surface area of 1051 m^2^ g^−1^—a value 2-3 times larger than that reported for BN aerogels (350 m^2^ g^−1^[9] and 431 m^2^ g^−1^[10]). Figure 7(c) shows the pore size distribution of the BN aerogel given by the Barrett-Joyner-Halenda (BJH) method with two peaks at 12 and 21.5 nm. The peak at 12 nm originates from the hollow BN nanotubes. This is consistent with the TEM results in Fig. 4. The other wide peak centered at 21.5 nm might come from the holes among the 3D network structure of the BN nanotubes and nanosheets. This result is further proof of the differences between our BN aerogels and other BN-based porous materials.

Figure 7(d) shows the mass density and BET specific surface area of BN aerogels as a function of the CVD growth time. The mass density (0.6 to 4.6 mg cm^−3^) and BET specific surface area (177 to 1051 m^2^ g^−1^) of the BN aerogels can easily be controlled across a large range by adjusting the growth time of the CVD (see Figure S3 in the Supplementary Information). The results provide a very simple to synthesize ultralight BN aerogels with various porous properties with the capacity to satisfy diverse applications. Some interesting features of the ultralight BN aerogels are shown in photographs in Figure S4 and the Supplementary Information. The results also indicate a road to ultralight BN aerogels at CVD growth times of 30 minutes (Fig. 7(a)). However, CVD growth times that are too short result in BN aerogels that are not suitable because the BN units are too weak to support the continuous 3D network structure of the BN aerogels.

## Discussion

**Figure 8:**
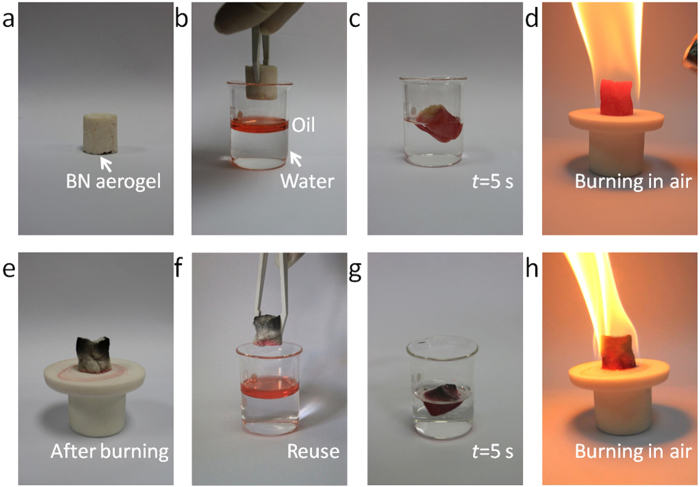
Photographs on oil absorption properties of a BN aerogel. **(a–d**) Absorption and burning process of cyclohexane (stained with Sudan Red II and floating on water) by the BN aerogel within 5 s. (**e–h**) BN aerogel after burning in air reused for absorption of cyclohexane.

Carbon aerogels are very promising materials for oil absorption^21^,^40^,^41^,^42^,^43^,^44^,^45^. Oil-saturated carbon aerogels can be regenerated for reuse via distillation or squeezing. Considering the similar structure of our BN aerogels as well as the carbon aerogel templates, the above process could also be employed to create BN aerogels for oil absorption and remediation. However, as our BN aerogels do not belong to structural materials, we usually pay more attention to the excellent performance of our BN aerogels during daily transportation, oil absorption and burning, which could also indicate the promising stiffness of the BN aerogels. Importantly, BN aerogels have oxidation resistance at much higher temperatures than carbon aerogels. Because of this, the BN aerogels can quickly be restored for reuse via simple burning in air (Fig. 8).

The oil absorption properties of a BN aerogel are illustrated with photographs in Fig. 8(a–c) show the as-synthesized BN aerogel (0.020 g) absorbing up to 160 times its own weight in cyclohexane (3.2 g, liquid, stained with Sudan Red II) while repelling water within 5 seconds. This value of oil absorption is 33-fold higher than porous BN nanosheets described previously^20^. Figure 8(d) shows the burning of the oil-saturated BN aerogel in air for cleaning, and Fig. 8(e) is the BN aerogel after burning. The BN aerogel turned partially black as a result of the carbon residue. This is common for organics burning in air. Subsequently, the cleaned BN aerogel with carbon residue was reused for a second oil absorption shown by Fig. 8(f–h). Cyclohexane (3.2 g) was absorbed by the cleaned BN aerogel within 5 seconds. The BN aerogel exhibits equal oil absorption properties after burning in air indicating its complete regeneration. Accordingly, the BN aerogel is an efficient and easy recyclable absorber for oils with applications in water treatment and environmental protection. It is especially relevant to high temperature oxidation conditions.

In summary, ultralight BN aerogels with a 3D porous network structure have been synthesized using template-assisted, catalyst-free, low-pressure CVD. The three-dimensional structure of the BN aerogels was achieved easily through the structural design of carbon aerogel templates. The mass density (0.6 to 4.6 mg cm^−3^) and BET specific surface area (177 to 1051 m^2^ g^−1^) of the synthesized BN aerogels can be easily controlled across a large range by adjusting the growth time of the CVD. The BN aerogels absorb up to 160 times their own weight in oil while repelling water. The BN aerogels after oil absorption can be restored for reuse simply by burning them air. This is straightforward because the oil burns off but the substrate has high resistance to this temperature increase. These features make this tool a promising material for heat protection, catalyst support, poison control, water purification, and environmental protection. Finally, the synthesis of BN aerogels using a template-assisted CVD technique could be extended to the synthesis of other ultralight porous materials.

## Methods

BN aerogels were grown in a 50 mm diameter quartz tube. Borazine was synthesized by the reaction of NaBH_4_ and (NH_4_)_2_SO_4_ and purified according to our previous reports^36^,^37^. Carbon aerogels (graphene-CNTs composite aerogels) were fabricated with commercial graphene and multi-walled CNTs using a published method^21^. The tube was heated to 900 °C before the growth of BN. The as-received carbon aerogels were then annealed for 30 minutes in an argon/hydrogen environment (Ar/H_2_, 5:1, 99.999% from Pujiang, China) at a flow of 270 sccm (standard cubic centimeters per minute), to remove residual impurities from the carbon aerogels. During this growth, borazine in a homemade bubbler was introduced to the growth chamber by another round of Ar flow of 5 sccm, while the Ar/H_2_ flow remained constant. Atomically thin BN coatings were deposited on the carbon aerogels via CVD to form BN/carbon aerogels with a low atmospheric pressure of 100 Pa and a typical growing time of 30 to 120 minutes. After this growth step, the BN/carbon aerogels were annealed in oxygen at 600 °C to remove the carbon aerogel templates. Hence, the BN aerogels were realized with the composite morphology of graphene-like BN nanosheets and CNTs-like BN nanotubes.

Raman spectroscopy was performed in a Thermo DXR with 532 nm laser excitation to show the lattice vibration modes of the aerogels. We measured the chemical composition of the aerogels with X-ray photoelectron spectroscopy (XPS) (AXIS Ultra^DLD^). Scanning electron microscopy (SEM) (Hitachi S4800) and field emission transmission electron microscope (TEM) (Tecnai G^2^ 20) were employed to observe the morphologies of the samples. Nitrogen adsorption isotherms and pore size distributions were measured at 77 K with a porosimeter (Micromeritics ASAP 2010). The Brunauer-Emmett-Teller (BET) specific surface area was calculated from the nitrogen adsorption data in relative pressures ranging from 0.05 to 0.30. The X-ray diffraction (XRD) (Bruker D8) was measured using Cu Kα radiation to show the structure of the aerogels. Fourier transformation infrared (FT-IR) spectroscopy and simultaneous thermal analysis of thermogravimetric analysis / differential scanning calorimetry (TGA/DSC) of the aerogels were performed using Nicolet 6700 and SDT Q600, respectively.

## Author Contributions

Y.S., B.L., G.D., S.Y. and C.Z. designed experiments. Y.S. and C.Z. carried out most experiments and measurements. B.L. synthesized borazine, and S.Y. prepared graphene-C.N.T.s composite aerogels. Y.S., B.L., G.D., C.Z. and X.X. discussed the results. Y.S. and C.Z. wrote the manuscript and all authors contributed to revisions.

## Additional Information

**How to cite this article**: Song, Y. *et al.* Ultralight boron nitride aerogels via template-assisted chemical vapor deposition. *Sci. Rep.* **5**, 10337; doi: 10.1038/srep10337 (2015).

## Supplementary Material

Supplementary Information
