# Supplementary material for: Ultralight boron nitride aerogels via template-assisted chemical vapor deposition
Source: Sci Rep. 2015 May 15;5:10337. doi: 10.1038/srep10337 (PMC4432566; doi:10.1038/srep10337)
Supplement: Supplementary Information [file srep10337-s1.doc]

**Supplementary Information**

**Ultralight boron nitride aerogels via template-assisted chemical vapor deposition**

**Authors:**

Yangxi Song^1,3^, Bin Li^1,★^, Siwei Yang^2^, Guqiao Ding^2,★^, Changrui Zhang^1,3^, Xiaoming Xie^2^

**Affiliations:**

^1^ Science and Technology on Advanced Ceramic Fibers and Composites Laboratory, College of Aerospace Science and Engineering, National University of Defense Technology, 109 Deya Road, Changsha 410073, PR China.

^2^ State Key Laboratory of Functional Materials for Informatics, Shanghai Institute of Microsystem and Information Technology, Chinese Academy of Sciences, 865 Changning Road, Shanghai 200050, PR China.

^3^ These authors contributed equally to this work.

^★^ e-mail: libin@nudt.edu.cn (B. Li); gqding@mail.sim.ac.cn (G. Ding)

Supplementary Figure S1


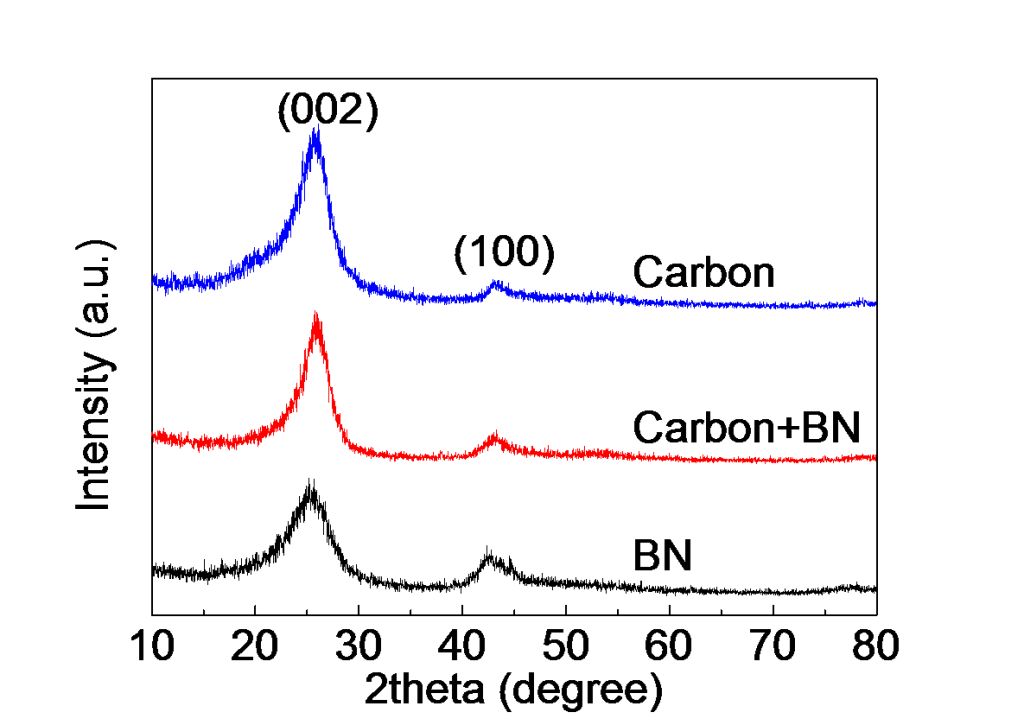


**Figure S1** Raman spectra of a carbon areogel template, a BN/carbon aerogel and a BN aerogel. However, the aerogels show similar XRD results, according to the extremely close crystal lattice parameters of carbon and BN. Wide peaks centered at about 2θ=26.5° and 2θ=42.5° are indexed as (002) and (100) plane of carbon or BN, respectively.

Supplementary Figure S2


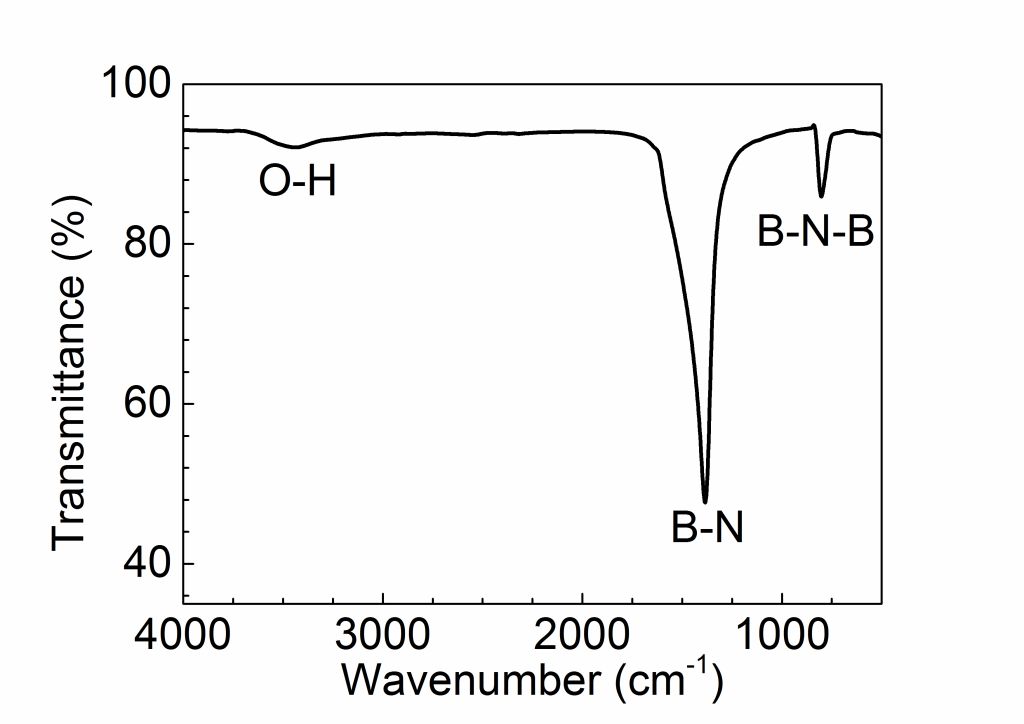


**Figure S2** Fourier transformation infrared (FT-IR) spectrum of a BN aerogel. The peak located at 1385 cm^-1^ is assigned to the in-plane stretching vibrations of B-N, whereas the peak at 809 cm^-1^ shows the B-N-B bending vibrations mode. The weak and broad peak centered around 3442 cm^-1^ is from O-H vibrations, which may originate from the water molecules absorbed by the sample during storage.

Supplementary Figure S3


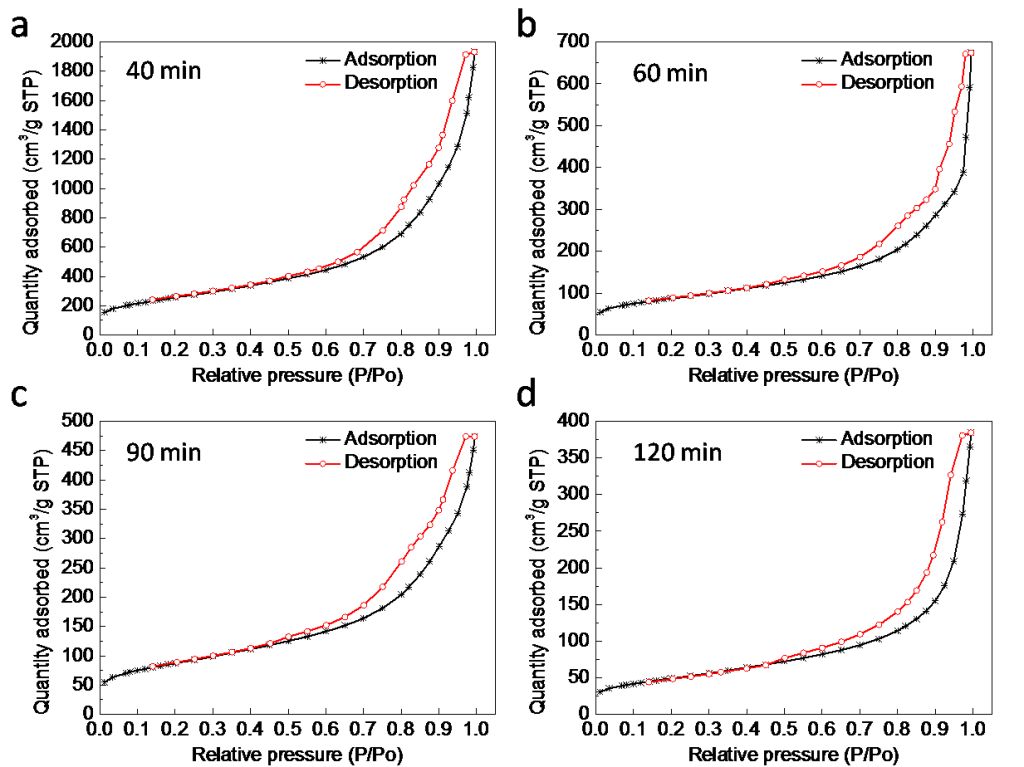


**Figure S3** N_2_ adsorption/desorption isotherms at 77 K of BN aerogels after different CVD growth time. **a**, 40 min. **b**, 60min. **c**, 90min. and **d**, 120min, with the corresponding BET specific surface area of 933 m^2^ g^-1^, 389 m^2^ g^-1^, 313 m^2^ g^-1^ and 177 m^2^ g^-1^, respectively.

Supplementary Figure S4

**
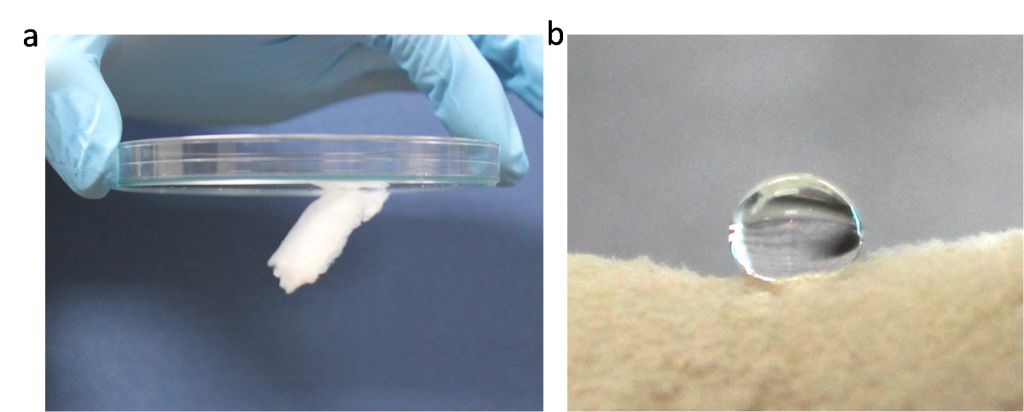
**

**Figure S4** Interesting photographs of BN aerogels. **a**, An ultralight BN aerogel attracted by a plastic dish as a result of static electricity. **b**, A tiny drop of water on surface of a BN aerogel.

Supplementary Figure S5


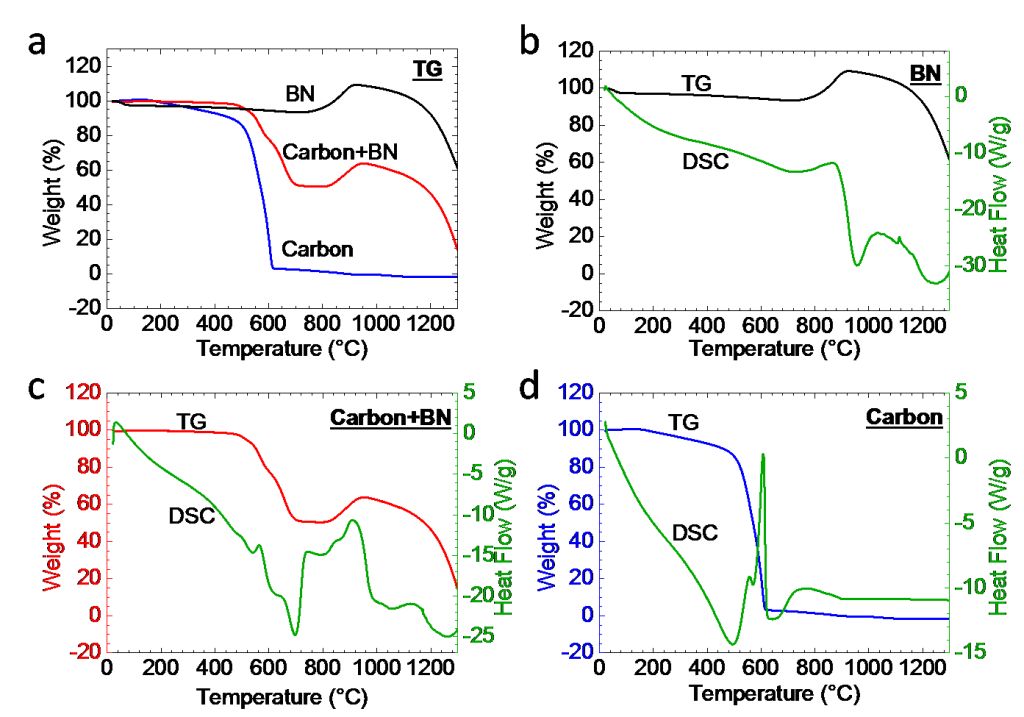


**Figure S5** Simultaneous TG/DSC analysis for aerogels in O_2_/N_2_ (1:4) from 0 to 1300 °C. The carbon aerogel shows weight loss as a result of oxidation, with nearly 0% weight residue over 600 °C. The BN aerogel exhibits slight weight increasement for the change from BN to B_2_O_3_, while with gradual weight loss for the sublimation of B_2_O_3_ at higher temperature, however with over 60% weight residue at 1300 °C.

Supplementary Figure S6


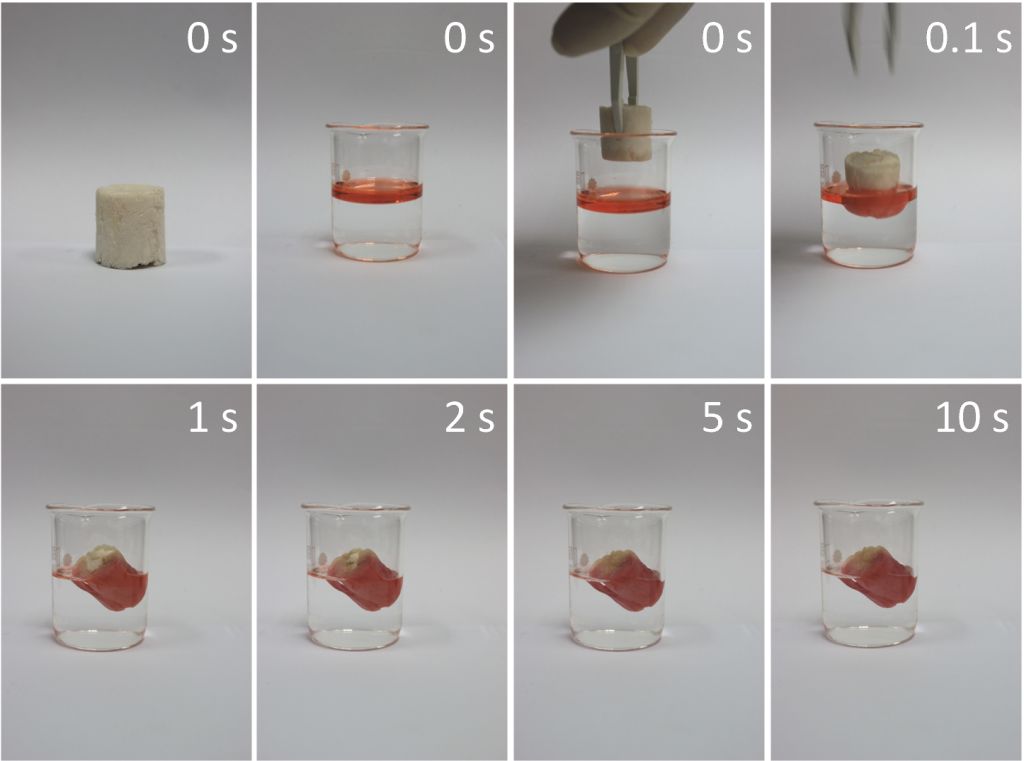


**Figure S6** Photographs on oil absorption process of a BN aerogel within 10 s. Cyclohexane (stained with Sudan Red II) on salt water was absorbed, with no obvious change occurring to the BN aerogel after 5 s, indicating the oil absorption has been finished within 5 s.
